# Dr. Robert Storr’s Case of Venous Hæmorrhage from an Abscess Resulting from Erysipelas Phlegmonoides

**Published:** 1842-06-25

**Authors:** Robert Storrs

**Affiliations:** Doncaster


*Case of Venous Hæmorrhage from an Abscess resulting from Erysipelas Phlegmonoides.* By ROBERT STORRS, Esq., Doncaster.—William Heaton, a strong muscular man, aged 36, by trade a master shoemaker, was seized, Dec. 14, 1839, with severe febrile symptoms, accompanied by an extensive diffused swelling over the front of the throat, affecting its cellular texture from the chin to the chest, of a phlegmonous erysipelatous character. In spite of leeches, fomentations, poultices, &c., and other local and general treatment, suppuration commenced, and on the 21st, evident fluctuation could be felt on the left side of the larynx. As the constitutional symptoms were very severe, I opened the abscess immediately, and above a pint of excessively offensive and sanious pus was discharged, accompanied with some large shreds of what appeared then to be dead cellular structure. From this opening, which was a very free one, an immense discharge took place, at a moderate computation, about a pint or more daily; and the sac of the abscess was found to extend itself from the left of the larynx and trachea, to the nape of the neck on that side, and from the angle of the jaw to the clavicle. The discharge of pus continued without abatement, and of a most offensive smell; severe symptoms of constitutional irritation and of exhaustion came on, and he required the constant administration of wine and of opiates.

On the 30th some hæmorrhage occurred from the opening, evidently of venous blood, which was controlled by pressure over the internal jugular, a little below the angle of the jaw; this pressure was kept up by mechanical means, and though the discharge of pus continued, no blood escaped until the night of the 1st of January, when it occurred, and was again stopped. During the following night, however, it again burst forth, and on my arrival I found him moribund. No examination was permitted after death, but there could be no doub t ut the abscess had implicated the internal jugular vein, the coats of which had been destroyed, and had been probably partly cast off at the time of the abscess being opened, and that a large discharge of blood had been then prevented by the deposition of coagulable lymph at the venous orifice.—*Provincial Medical and Surgical Journal.* April 2, 1842.

[Cases of considerable hæmorrhage from the progress of diffuse inflammation of the cellular tissue are very rare, though the great vessels are not unusually isolated by the sloughing of the free tissue around them, sometimes to the extent of several inches. Our memory supplies us with but one case of somewhat analogous character. It occurred in the Pennsylvania Hospital, many years ago. The patient had recently recovered from a protracted fever when he met with a compound fracture of the leg, succeeded by erysipelas phlegmonoides. While engaged some days after the admission in dressing the limb, in which there were a number of orifices, we were shocked by the occurrence of a torrent of hæmorrhage bursting instantaneously and in full tide through all the openings simultaneously. Both venous and arterial currents were distinctly noticed. No large vessel was implicated in the original accident, and the bleeding could not have been the result of the opening of either of the principal arteries or veins of the limb, for it was at once too profuse and too widely diffused to admit of this supposition. Three or four seconds could scarcely have elapsed before the hæmorrhage was checked by the pressure of the thumb upon the femoral artery in the groin, yet the loss of blood was so tremendous that the patient succumbed in a few hours, it having been decided that amputation was out of the question, in consequence of excessive debility. Might not the hæmorrhage in the case of Mr. Storrs have been derived from the smaller vessels implicated in the sloughs, rather than from the jugular vein? We have seen the femoral vessels, through the usual cutaneous orifices in erysipelas phlegmonoides, totally detached in the gaping cavity from which the sloughs had been drawn and the pus expelled by pressure; and they were traced in this condition for many inches, yet the patient recovered without hæmorrhage.

In some forms of gangrene which are not confined, like the disease just mentioned, to one particular tissue, the great vessels are more frequently involved in the sloughs without being previously obliterated. It happened to us once to lose a child about ten years of age, by the rupture of the carotid artery from the gangrene attendant upon putrid sore throat. R. C.]

